# Initiation, cessation and relapse of tobacco smoking over a 3-year period among participants aged ≥15 years in a large longitudinal cohort in rural South Africa

**DOI:** 10.1371/journal.pgph.0004126

**Published:** 2025-02-25

**Authors:** Ronel Sewpaul, Stephen Olivier, Hloniphile Ngubane, Thando Zulu, Mareca Sithole, Vukuzazi Team, Willem A. Hanekom, Gina Kruse, Nancy A. Rigotti, Mark J. Siedner, Emily B. Wong, Krishna P. Reddy

**Affiliations:** 1 Public Health, Societies and Belonging, Human Sciences Research Council, Cape Town, South Africa; 2 Africa Health Research Institute, KwaZulu-Natal, South Africa; 3 Division of General Internal Medicine, School of Medicine, University of Colorado, Denver, Colorado, United States of America; 4 Tobacco Research and Treatment Center, Massachusetts General Hospital, Boston, Massachusetts, United States of America; 5 Department of Medicine, Harvard Medical School, Boston, Massachusetts, United States of America; 6 Medical Practice Evaluation Center, Massachusetts General Hospital, Boston, Massachusetts, United States of America; 7 Division of Infectious Diseases, Heersink School of Medicine, University of Alabama at Birmingham, Birmingham, Alabama, United States of America; 8 Division of Pulmonary and Critical Care Medicine, Massachusetts General Hospital, Boston, Massachusetts, United States of America; International Medical University, MALAYSIA

## Abstract

Tobacco smoking is increasing in many low-and-middle-income countries, but data about initiation and cessation patterns are sparse, particularly in rural areas. We investigated changes in smoking status and their determinants in rural South Africa. Participants enrolled in the Vukuzazi population cohort in rural KwaZulu-Natal, South Africa completed a baseline tobacco behavioural survey during 1 May 2018 to 31 March 2020. A follow-up survey was conducted during 4 May 2021 to 18 November 2022 among all participants aged ≥15 years who reported current and former smoking at baseline (to detect cessation and relapse) and in a random selection of participants aged 15–29 years who reported never smoking at baseline (to detect initiation). We fit regression models to estimate smoking initiation (from never to current or former smoking), cessation (from current to former smoking) and relapse (from former to current smoking) between baseline and follow-up, and to investigate the sociodemographic and behavioural variables associated with each outcome. Of those recruited, 52% (754/1448) participated in the follow-up survey, which occurred a median of 3.0 years (IQR: 2.6–3.2) from baseline. Initiation, cessation and relapse occurrence was 12.0% (95% CI: 8.4–16.8), 12.9% (95% CI: 10.0–16.5) and 10.9% (95% CI: 4.4–24.2), respectively. Males had significantly higher odds of initiation than females (adjusted odds ratio [AOR] 12.81, 95% confidence interval [CI]: 3.54–46.36). Moderate/heavy smoking (≥10 products per day; AOR 0.27, 95% CI: 0.08–0.93 relative to light smoking <10 products per day) and middle socioeconomic status (AOR 0.37, 95% CI: 0.15–0.89 relative to low socioeconomic status) were associated with lower odds of cessation. No covariates were significantly associated with relapse. In conclusion, most people retained their smoking status over approximately three years in rural South Africa. Fewer than one in eight smokers quit. Prevention interventions are needed to address high initiation among young males. People who smoke moderately or heavily and people with middle socioeconomic status may benefit from targeted cessation interventions.

## Introduction

Tobacco use remains one of the largest public health threats globally, with over 8 million deaths attributable to smoking annually, in addition to immense health systems burden and economic costs [1]. Four in five tobacco users live in low- and middle-income countries (LMICs) [2], which carry a high burden of tobacco-related morbidity and mortality [1,3]. South Africa, a middle-income country, experienced significant declines in tobacco smoking [4,5] following the implementation of the Tobacco Products Control Amendment Act in 1999 [6] and its subsequent amendments [7,8]. However, increases in tobacco use have been reported in recent years [9]. The Global Adult Tobacco Survey 2021 showed that a quarter of South Africans aged 15 years and older smoke tobacco (41.2% among males and 11.5% among females) [10], higher than reported in the 2016 South African Demographic and Health Survey (SADHS) (37% among males and 8% among females) [4].

The prevalence of tobacco smoking depends on smoking initiation, cessation, and relapse rates [11]. Quantifying these rates and understanding their determinants is key to informing tobacco control strategies and model projections of the impact of changes in tobacco use over time. Studies of the changes in smoking status over time and their determinants are scarce in South Africa. These data are needed to inform how to design targeted tobacco control interventions, including tailoring interventions for the highest impact for sub-populations at risk for initiation, cessation, and relapse. Using data from the Vukuzazi population cohort in rural KwaZulu-Natal in South Africa [12], an area with high HIV burden, we investigated smoking initiation, cessation and relapse rates over an approximately 3-year period and their associated sociodemographic and behavioural determinants.

## Materials and methods

### Study design

The Vukuzazi programme conducted community-based health phenotyping within the uMkhanyakude district in rural KwaZulu-Natal, South Africa [12]. The programme was established in 2018 to determine the prevalence and intersection of infectious and non-communicable diseases in the population. It was a nested closed cohort of the Population Intervention Programme (PIP) [13] and was conducted in the southern part of the PIP area. The area is characterized by high HIV prevalence and high unemployment rates [12]. 18,041 people aged 15 years and older were enrolled in the Vukuzazi programme, corresponding to approximately half of the eligible population of this age range. Of these participants, 34.2% were living with HIV [12]. Baseline data collection was conducted in-person at a mobile health camp during 1 May 2018 to 31 March 2020, and included a nurse-administered survey on personal history of tobacco and alcohol use, HIV, tuberculosis (TB), non-communicable diseases and quality of life, as well as measurement of anthropometric factors, blood pressure, and various clinical biomarkers. Further information on the Vukuzazi cohort is detailed elsewhere [12].

### Tobacco use sub-study sampling frame

During 4 May 2021 to 18 November 2022, a follow-up telephonic tobacco-focused survey was conducted among a subset of Vukuzazi participants to assess the changes in their smoking behaviours over time. The follow-up study intended to enroll purposive samples of approximately 1000 individuals aged 15 years and older in the baseline Vukuzazi study who had reported current (n = 1301) smoking at that time, 100 individuals aged 15 years and older at baseline who reported former (n = 150) smoking at that time and a random sample of 400 individuals aged 15–29 years at baseline who had reported never smoking (n = 6184) at that time, in order to estimate smoking cessation, relapse and initiation. We chose the more limited age range for “never smoking” because few people in South Africa initiate smoking after age 29 years [14,15]. We aimed to contact an equal number of people with HIV and people without HIV within each smoking status category.

### Data collection

Prospective participants for the follow-up survey were contacted telephonically. The survey was available to complete in English or isiZulu, the latter being the prominent language spoken in the study area. The questionnaire was translated from English to isiZulu, and then back-translated to check for accuracy. Survey administration was conducted by nurses who are bilingual in English and isiZulu. For each prospective participant, up to six contact attempts were made. Participants who reported current tobacco smoking during the follow-up survey and who expressed interest in quitting or reducing their smoking were offered telephonic counselling and were referred to a quit line at the end of the follow-up survey, of which the details are described elsewhere [16].

### Measures

#### Smoking status outcome measures.

The tobacco items in the baseline survey were based on the WHO STEPS questionnaire [17]. The tobacco items in the follow-up survey, which included more details about specific tobacco smoking behaviours, were based on the U.S. National Health Interview Survey [18]. In the baseline survey, people who currently smoked (CS) were defined as participants who reported smoking any tobacco products, such as cigarettes, cigars or pipes. Participants were shown a card that illustrated the products defined as smoking tobacco in South Africa. People who formerly smoked (FS) were participants who reported having ever smoked any tobacco products in the past but not currently. People who never smoked (NS) were participants who reported that they were not currently smoking and had never smoked tobacco products. In the follow-up survey, CS were defined as having smoked 100 tobacco products, such as cigarettes, cigars, hand-rolled cigarettes, or pipes, in their lifetime and who currently smoked tobacco [18]. FS were participants who had smoked at least 100 tobacco products in their lifetime and who had quit smoking. NS were those who had never smoked or smoked less than 100 tobacco products in their lifetime. The 100 tobacco product criterion is often used in tobacco surveys to define ever smoking [19].

The three primary outcome measures in this study were initiation, cessation and relapse of tobacco smoking. These were derived by assessing the changes in smoking status between baseline and follow-up surveys. Smoking initiation among 15–29-year-olds was derived from baseline NS who reported being CS or FS at follow-up. Smoking cessation was defined by baseline CS who reported being FS at follow-up. Relapse was defined by baseline FS who reported CS at follow-up.

#### Covariates.

Covariate selection was based on a literature review of the factors associated with smoking initiation, cessation and relapse [14,20–30] and a review of the available data per smoking outcome. Covariates for the outcome of smoking initiation were baseline age, sex, employment, socioeconomic status (SES), HIV care cascade status and past-year alcohol use. Covariates for the outcome of smoking cessation included baseline age, sex, employment, SES, years since starting smoking, smoking intensity, having made a quit attempt during the past year, having been advised by a health care provider to quit smoking, HIV care cascade status, past-month alcohol use, and diagnoses of hypertension and diabetes; as well as self-reported diagnosis of incident TB between baseline and follow-up and difficulties in carrying out daily activities that were both measured at follow-up. For the outcome of smoking relapse, baseline age, HIV status (positive or negative), SES and past-year alcohol use were used as covariates.

Smoking intensity was derived from the total numbers of combustible tobacco products used on a typical day and was categorized into light (<10 products per day), moderate (10–19) and heavy (≥20) smoking, where the latter two groups were combined due to the small numbers of heavy smokers with data on quitting outcomes. HIV care cascade status was categorized as HIV negative, HIV positive and uncontrolled (either undiagnosed, diagnosed and not in care, or in care and viral load [VL] ≥400 copies/ml) or HIV positive and controlled (diagnosed, in care and VL <400 copies/ml). Further details on the definition of the covariates are presented in S1 Table.

### Sample size

Allowing for at least a 67% response rate, we expected to enroll approximately 1000 CS and 100 FS. These samples would allow us to estimate 10% cessation rate with a precision of 8–12% and 10% relapse with a precision of 5–18%. A sample of 400 NS aged 15–29 years would allow us to estimate 10% initiation with a precision of 8–13%. We aimed for an equal split between people with and without HIV.

### Statistical analysis

Analyses were performed using Stata version 15.0 (Stata Corp, College Station, Texas, USA). Descriptive statistics (percentages and counts) were presented for the cumulative incidence of smoking initiation, cessation and relapse between the baseline and follow-up surveys. Differences in proportions by sex and age group were assessed using Pearson Chi-square tests and Fisher’s exact tests. Univariate logistic regressions were first used to assess the variables associated with smoking initiation, cessation and relapse. When there were small numbers of observations for categories of covariates for an outcome (n < 8), those covariates could not be included in the regression models for that outcome. Variables with a p-value of <0.25 in univariate logistic regression models were included in the multivariable regression models [31]. Results were expressed as crude and adjusted odds ratios (OR and AOR) with 95% confidence intervals. Statistical significance was assessed with p < 0.05. Average adjusted predicted probabilities of initiating and quitting smoking were calculated using the *margins* commands in Stata.

In primary analyses, inconsistent records of participants who reported being CS at baseline and NS at follow-up (n = 47) were excluded from the calculation of the smoking cessation variable. Similarly, participants who reported being FS at baseline and NS at follow-up (n = 16) were excluded from the calculation of the relapse variable. Sensitivity analyses were therefore conducted, where the inconsistent records were included in the denominator and numerator of the cessation variable and in the denominator of the relapse variable, and the analyses were repeated to determine their influence on the results.

The missing values for employment status among all participants (13.9% missing) and for smoking intensity and years since started smoking among baseline current smokers (16.1% and 19.5% missing) were coded into a separate category called ‘unknown’. Missingness was 2.9% for SES and <1% for daily difficulties, hypertension, diabetes and incident TB. Multiple imputation was not performed because the data were assumed to be missing completely at random after a data exploration showed no correlations >0.20 between smoking intensity, years since started smoking and the other variables (S2 Table).

### Ethical approval

Ethical approval for the study was obtained from the Ethics Committees of the University of KwaZulu-Natal (BE560/17), London School of Hygiene & Tropical Medicine (#14722), the Mass General Brigham Institutional Review Board (2018P001802) and University of Alabama at Birmingham (#300007237). Written informed consent (and parent/guardian consent for all unemancipated individuals 15–17 year of age) was obtained at baseline from all participants, including consent to be contacted for future follow-up studies, as described previously [12,32]. For the follow-up tobacco survey, verbal consent (telephonic) for participation was obtained before proceeding to the survey questions. Participants were informed of voluntary participation, the anonymity and confidentiality of their responses, and the right to withdraw from the survey at any time.

### Inclusivity in global research

Additional information regarding the ethical, cultural, and scientific considerations specific to inclusivity in global research is included in the Supporting information (S1 Checklist).

## Results

### Follow-up survey participation

**Fig 1:**
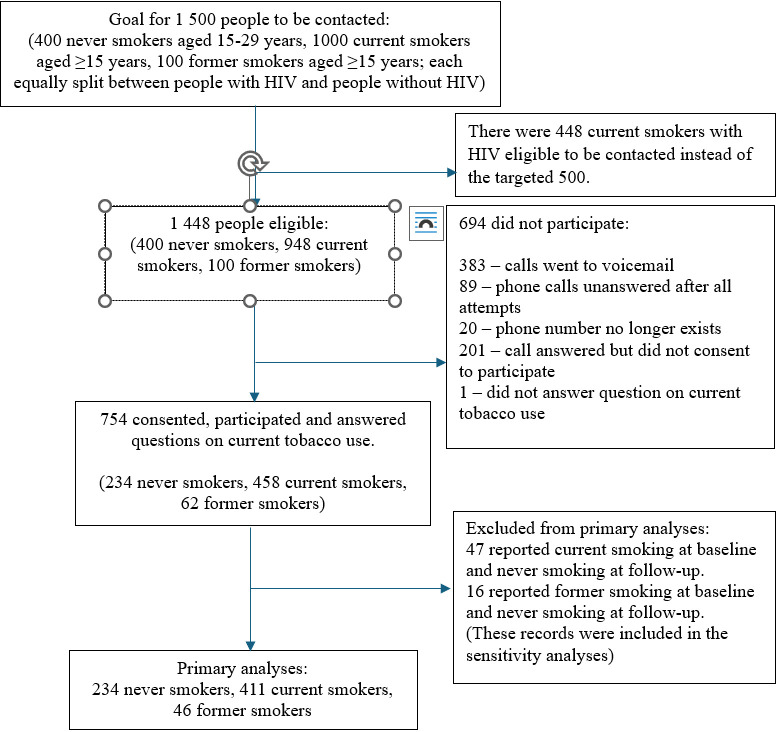
Participant flowchart.

Only 448 participants in the baseline cohort were CS with HIV, short of the anticipated goal of 500. Therefore, instead of 1500, a total of 1448 participants were eligible for the follow-up survey, comprising 948 baseline CS and 100 baseline FS aged ≥15 years and 400 baseline NS aged 15–29 years. Of the 1448 people who were eligible, 754 (52.1%) were successfully reached and consented to the follow-up survey and answered the questions on smoking status (Fig 1). Response rates for baseline CS, FS and NS were 48.3% (n = 458/948), 62.0% (n = 62/100) and 58.5% (n = 234/400), respectively. Participants in the follow-up survey included a lower proportion of males (74.9%) than those who did not participate in the follow-up survey (80.0%) (p = 0.022) but did not differ significantly with respect to age, HIV status, employment and SES (S3 Table).

Forty-seven participants reported being CS at baseline and NS at follow-up (10.3% of all baseline CS) and 16 reported being FS at baseline and NS at follow-up (25.8% of all baseline FS). These inconsistent records were excluded from the primary analyses. Hence, 411 CS, 46 FS and 234 NS were included in the primary analyses. The median time between the baseline and follow-up surveys was 3.0 years (IQR: 2.6–3.2).

### Changes in smoking status

Over 87% of participants retained their smoking status between baseline and follow-up (Table 1). Twelve percent of baseline NS initiated smoking (12%, 95% CI: 8.4–16.8), with nearly tenfold higher smoking initiation among males (22.5%, 95% CI: 15.7–31.3) than females (2.4%, 95% CI: 0.8–7.3) (p < 0.001). Smoking initiation among males was 29.0% (95% CI: 19.1–41.5) for 20–29-year-olds and 14.3% (95% CI: 6.9–27.1) for 15–19-year-olds (p = 0.066). Smoking cessation (among baseline CS) was 12.9% (95% CI: 10.0–16.5) (males: 12.3%, 95% CI: 9.4–16.0, females: 28.6%, 95% CI: 11.1–56.2) and smoking relapse (among baseline FS) was 10.9% (95% CI: 4.4–24.2) (males: 12.2%, 95% CI: 5.0–26.9, females: 0.0%).

**Table 1:** Changes in smoking status between baseline and follow-up.

	Smoking initiation (among baseline NS)^1^				Smoking cessation (among baseline CS)^2^				Smoking relapse (among baseline FS)^2^			
	%	95% CI	n	p-value	%	95% CI	n	p-value	%	95% CI	n	p-value
Total	12.0	8.4–16.8	28/234		12.9	10.0–16.5	53/411		10.9	4.4–24.2	5/46	
Male	22.5	15.7–31.3	25/111	<0.001	12.3	9.4–16.0	49/397	0.092	12.2	5.0–26.9	5/41	1.000
Female	2.4	0.8–7.3	3/123		28.6	11.1–56.2	4/14		0.0	–	0/5	
Males 15–19 years	14.3	6.9–27.1	7/49	0.066								
Males 20–29 years	29.0	19.1–41.5	18/62									

^1^Among participants 15–29 years old.

^2^Among participants ≥15 years old.

### Determinants of changes in smoking status

Tables 2–4 show the results of the logistic regression models for initiation, cessation and relapse among baseline NS, CS and FS, respectively. Among baseline NS aged 15–29 years, males had significantly higher odds of initiating smoking than females (AOR 12.81, 95% CI: 3.54–46.36). S1 Fig. presents the marginal probabilities of initiating smoking by sex. Participants living with HIV who were either undiagnosed, diagnosed and not in care, or in care and with a viral load ≥400 copies/ml had higher crude odds of smoking initiation than participants living with HIV who were in care and with undetectable viral load (OR 2.98, 95% CI: 1.24–7.18) but this was not statistically significant in the adjusted model.

**Table 2:** Logistic regression results showing variables associated with smoking initiation among people who reported never smoking at baseline, ages 15–29 years.

	%	n	Number who initiated smoking	Univariate logistic regression			Multiple logistic regression		
				OR	95% CI (OR)	p-value	AOR	95% CI (OR)	p-value
Sex									
Male	47.4	111	25	11.63	[3.40–39.75]	<0.001	12.81	[3.54–46.36]	<0.001
Female	52.6	123	3	ref	–		ref	–	
Age at baseline survey									
15–19 years	33.8	79	7	ref	–	–			
20–29 years	66.2	155	21	1.61	[0.65–3.97]	0.300			
HIV care cascade status									
Negative	50.0	117	11	ref	–	–	ref	–	–
Positive and uncontrolled^1^	23.5	55	13	2.98	[1.24–7.18]	0.015	2.11	[0.77–5.79]	0.147
Positive and controlled^2^	26.5	62	4	0.66	[0.2–2.18]	0.500	0.85	[0.23–3.17]	0.808
Consumed alcohol in past 12 months									
Yes	10.3	24	1	0.29	[0.04–2.27]	0.241	0.15	[0.02–1.26]	0.080
No	89.7	210	27	ref	–	–	ref	–	–
Employment status									
Employed	9.8	23	5	ref	–	–	ref	–	–
Unemployed	42.3	99	14	0.59	[0.19–1.86]	0.369	0.74	[0.19–2.93]	0.673
Not in labour force	15.8	37	2	0.21	[0.04–1.17]	0.074	0.26	[0.04–1.78]	0.168
Unknown	32.1	75	7	0.37	[0.11–1.31]	0.122	0.28	[0.06–1.25]	0.094
Socioeconomic status^3^									
Low	41.9	96	12	ref	–	–			
Middle	22.3	51	5	0.76	[0.25–2.29]	0.627			
High	35.8	82	11	1.08	[0.45–2.61]	0.856			

OR, odds ratio; AOR, adjusted odds ratio; CI, confidence interval.

^1^HIV positive and either undiagnosed, diagnosed and not in care, or in care and viral load [VL] ≥400 copies/ml.

^2^HIV positive and diagnosed, in care and VL <400 copies/ml.

^3^Socioeconomic status was measured by an asset-based index derived from a standard list of questions about household items, water source, toilet type and electricity source. The index was categorized into quintiles corresponding to low, medium and high socioeconomic status.

**Table 3:** Logistic regression results showing variables associated with smoking cessation among people who reported currently smoking at baseline, ages ≥15 years.

	%	n	Number who quit smoking	Univariate logistic regression			Multiple logistic regression		
				OR	95% CI (OR)	p-value	AOR	95% CI (AOR)	p-value
Sex									
Male	96.6	397	49	0.35	[0.11–1.17]	0.087	0.28	[0.08–1.02]	0.054
Female	3.4	14	4	ref	–	–	ref	–	
Age at enrolment									
15–49 years	72.7	299	37	ref	–	–			
≥50 years	27.3	112	16	1.18	[0.63–2.22]	0.607			
HIV care cascade status									
Negative	50.6	208	29	ref	–	–			
Positive and uncontrolled^1^	13.1	54	5	0.63	[0.23–1.71]	0.365			
Positive and controlled^2^	36.3	149	19	0.90	[0.48–1.68]	0.745			
Incident tuberculosis									
Yes	8.1	33	7	1.93	[0.79–4.69]	0.149	1.46	[0.56–3.77]	0.437
No	91.9	375	46	ref	–	–			
Daily difficulties									
None	88.5	361	48	ref	–	–			
Some	11.5	47	5	0.78	[0.29–2.06]	0.611			
Consumed alcohol in past 30 days									
Yes	28.2	116	14	0.90	[0.47–1.73]	0.754			
No	71.8	295	39	ref	–	–			
Employment status									
Employed	30.2	124	14	ref	–	–	ref	–	–
Unemployed	56.2	231	27	1.04	[0.52–2.06]	0.911	0.86	[0.42–1.75]	0.669
Not in labour force	9.5	39	6	1.43	[0.51–4.01]	0.498	1.05	[0.36–3.08]	0.934
Unknown	4.1	17	6	4.29	[1.37–13.40]	0.012	5.36	[1.51–19.10]	0.010
Socioeconomic status^3^									
Low	37.3	148	25	ref	–	–	ref	–	–
Middle	25.2	100	8	0.43	[0.18–0.99]	0.048	0.37	[0.15–0.89]	0.027
High	37.5	149	19	0.72	[0.38–1.37]	0.317	0.61	[0.30–1.24]	0.174
Hypertension diagnosis									
Yes	20.0	82	13	1.36	[0.69–2.67]	0.379			
No	80.0	328	40	ref	–	–			
Diabetes diagnosis									
Yes	2.2	9	1	0.84	[0.10–6.85]	0.870			
No	97.8	401	52	ref	–	–			
Smoking intensity at baseline									
Light	65.7	270	36	ref	–	–	ref	–	–
Moderate to heavy	18.2	75	4	0.37	[0.13–1.06]	0.065	0.27	[0.08–0.93]	0.038
Unknown	16.1	66	13	1.59	[0.79–3.21]	0.192	1.64	[0.78–3.44]	0.190
Years since started smoking									
1–5 years	11.7	48	9	ref	–	–	ref	–	–
>5 years	69.2	283	35	0.61	[0.27–1.37]	0.232	0.92	[0.38–2.24]	0.847
Unknown	19.1	78	9	0.57	[0.21–1.54]	0.265	0.76	[0.26–2.22]	0.616
Attempted to quit smoking (past 12 months)									
Yes	14.4	59	7	0.90	[0.38–2.09]	0.799			
No	85.6	352	46	ref	–	–			
Advised to quit smoking by a health care provider									
Yes	5.8	24	2	0.60	[0.14–2.62]	0.496			
No	94.2	387	51	ref	–	–			

OR, odds ratio; AOR, adjusted odds ratio; CI, confidence interval.

^1^HIV positive and either undiagnosed, diagnosed and not in care, or in care and viral load [VL] ≥400 copies/ml.

^2^HIV positive and diagnosed, in care and VL <400 copies/ml.

^3^Socioeconomic status was measured by an asset-based index derived from a standard list of questions about household items, water source, toilet type and electricity source. The index was categorized into quintiles corresponding to low, medium and high socioeconomic status.

**Table 4:** Logistic regression results showing variables associated with smoking relapse among people who reported formerly smoking at baseline, ages ≥15 years.

	%	n	Number who relapsed smoking	Univariate logistic regression			Multiple logistic regression		
				OR	95% CI (OR)	p-value	AOR	95% CI (AOR)	p-value
Age at enrolment									
15–49 years	37.0	17	3	ref	–	–			
≥50 years	63.0	29	2	0.35	[0.05–2.32]	0.274			
HIV status									
Positive	45.7	21	1	ref	–	–	ref	–	–
Negative	54.3	25	4	3.81	[0.39–37.07]	0.249	4.64	[0.42–50.76]	0.209
Consumed alcohol in past 12 months									
No	69.6	32	4	ref	–	–			
Yes	30.4	14	1	0.54	[0.05–5.31]	0.596			
Socioeconomic status^1^									
Low	41.3	19	1	ref	–	–	ref	–	–
Middle	23.9	11	3	6.75	[0.61–75.27]	0.121	8.79	[0.73–106.41]	0.088
High	34.8	16	1	1.20	[0.07–20.85]	0.900	1.81	[0.10–33.91]	0.691

OR, odds ratio; AOR, adjusted odds ratio.

^1^Socioeconomic status was measured by an asset-based index derived from a standard list of questions about household items, water source, toilet type and electricity source. The index was categorized into quintiles corresponding to low, medium and high socioeconomic status.

Among baseline CS, moderate to heavy smoking (AOR 0.27, 95% CI: 0.08–0.93 compared to light smoking) and middle SES (AOR 0.37, 95% CI: 0.15–0.89 compared to low SES) were associated with lower odds of quitting smoking. CS with unknown employment status had higher odds of quitting smoking compared to those who were employed (AOR 5.36, 95% CI: 1.51–19.10) (Table 3). The average adjusted predicted probabilities of smoking cessation, after adjusting for other variables in the model, were 0.13 (95% CI: 0.09–0.16) for males and 0.32 (95% CI: 0.08–0.55) for females (S2 Fig). Moderate to heavy smokers had a 0.04 average adjusted probability of cessation. No covariates were significantly associated with relapse among FS (Table 4).

### Sensitivity analyses

When including the baseline CS and FS who reported being NS at follow-up in the denominators of the cessation and relapse variables, the prevalence of cessation and relapse were 11.6% (95% CI: 8.9–14.9) and 8.1% (95% CI: 3.3–18.3), respectively. As with the primary analyses, lower odds of cessation among baseline CS were found for moderate to heavy smokers compared to light smokers and for middle SES compared to low SES, but these results were not statistically significant (S4 Table). The finding of significantly higher odds of cessation among baseline CS with unknown employment status was confirmed in the sensitivity analysis. Among baseline FS, similar directions of associations were found between the covariates and relapse as in the primary analysis, and the finding of no significant associations was confirmed (S5 Table). When we included the baseline CS who reported NS at follow-up in both the numerators and denominators of the cessation variable, the prevalence of cessation was 21.8% (95% CI: 18.3–25.9). We found similar patterns of associations with cessation, with the addition of significantly higher odds of smoking cessation for females (S6 Table).

## Discussion

We investigated rates of smoking initiation, cessation and relapse in a prospective population-based cohort in rural South Africa. We found that, while the majority of participants retained their smoking status over the approximately 3-year period, smoking cessation and relapse occurred in 12.9% and 10.9% of participants, respectively. Smoking initiation occurred among 12% of people aged 15–29 years and was nearly tenfold higher for males than females. CS with moderate or high intensity smoking and from middle SES backgrounds were less likely to quit smoking. Tobacco control is crucial in high HIV burden settings, due to tobacco smoking being associated with worse outcomes among people with HIV including higher TB risk [33,34]. However, we found no significant associations between HIV status and changes in smoking status over time in this high HIV burden setting.

Smoking onset usually occurs during adolescence or early adulthood [14,15,24,35]. Tobacco prevention initiatives are vital for young people because those who start smoking at younger ages are more likely to smoke regularly and have lower chances of quitting than those who start at older ages [35]. More than one in five young males initiated smoking during the study period, with nearly tenfold higher smoking initiation among males than females. These findings are indicative of the high smoking prevalence among males in South Africa [4,10]. Other studies have confirmed higher smoking initiation among males than females [36,37]. Interventions to prevent smoking initiation should therefore focus heavily on boys and young men and be tailored to the differential determinants of smoking by gender [38,39]. Tobacco price increases, for example, led to reduced onset of regular smoking initiation in South African males but not in females [14]. Youth are also more sensitive to tobacco prices than older adults [40,41], suggesting that excise tax increases on tobacco products have potential to reduce smoking initiation in young males. In addition, the minimum purchase age policy of 18 years should be enforced at all points of sale and the purchasing age could be raised to 21 years. Other predictors of smoking initiation such as being unemployed and of lower socioeconomic status [24] were not confirmed in this study, which could be attributed to the low sample sizes for these groups.

While we found a smoking cessation rate of 12.9% among baseline CS, a cross-sectional study of South African smokers who had tried to quit reported a 14% quit rate [29]. Another study found that 7% of CS quit smoking during the 20-week tobacco sales ban during the COVID-19 lockdown in 2020 [42]. However, that study did not follow participants over time to assess changes in quit and relapse rates long after the ban.

Our finding of low-intensity smokers being more likely to quit has also been shown in other studies [23,25]. High-intensity smoking increases nicotine dependence, which is associated with reduced odds of successfully quitting smoking [20,21,28]. One South African study showed that people with high nicotine dependence are less likely to attempt to quit, while another study using 1998 SADHS data contradictorily showed that South Africans who smoke over 20 cigarettes daily were more likely to successfully quit than light smokers [29]. More intensive cessation interventions would be required for smokers with high nicotine dependence, including improved access to medications to support smokers to cease.

People of relatively lower SES, as measured by household assets and services, who smoke had higher likelihood of smoking cessation than those of middle SES. Low material wealth was also associated with increased cessation in India [43] while studies in some LMICs have found no clear associations between SES and smoking cessation [11]. Socioeconomic status can determine relative affordability of tobacco products. South Africa continued to increase tobacco product prices [44,45], and low SES individuals may be more responsive to price increases [46], which increases their likelihood of quitting.

This study is subject to some limitations. Firstly, the response rate in the follow-up survey was lower than expected, which could be due to the use of a telephonic rather than an in-person survey and the high levels of migration in the target population [47]. The low realized samples of CS, NS and FS affected the statistical power to detect significant differences in smoking status by the covariates of interest. Secondly, the survey did not measure all predictors that have been shown to be significantly associated with changes in smoking status such as risk perceptions about smoking, education level and exposure to smoke-free environments [23,26,27]. Thirdly, the measurement of cessation relied on self-report and was not biochemically verified. Fourthly, different questions were used to determine smoking status between baseline and follow-up, where the latter asked about lifetime smoking of at least 100 tobacco products. The inconsistent reporting by participants of CS or FS at baseline but NS at follow-up may have been a result of the different questions asked. These inconsistencies may also be due to social desirability bias in reporting never smoking. Moreover, some smokeless tobacco users may have reported smoking tobacco products at baseline because a specific question on smokeless tobacco use was not included at baseline. Finally, survey administration methods were different between the two surveys, where the baseline survey was administered in-person and the follow-up survey was administered telephonically. Telephone administration was used because South Africa was implementing COVID-19 lockdown measures during 2021 and, even when they were lifted, some households were reluctant to let data collectors into their homes.

The rates of smoking initiation, cessation and relapse can inform model projections of changes in tobacco use and tobacco use outcomes over time among rural South Africans [48]. More longitudinal studies are needed to assess changes in smoking status over time both in other LMICs and among South Africans in other geographical areas, in urban areas and in various race and SES groups. These data can help inform what types of tobacco control interventions to use and the target populations.

## Conclusions

We found high rates of smoking initiation and low rates of smoking cessation, especially among males, over a 3-year period in rural South Africa. Understanding tobacco use behaviours over time in a population can inform targeted or tailored interventions to prevent initiation and promote cessation. The high rates of smoking initiation, particularly among young males, demonstrates the need for smoking prevention interventions. Furthermore, higher smoking intensity and middle SES were associated with lower smoking cessation. Intensive cessation treatment programs could help moderate to heavy smokers to quit. Tailored interventions are needed to elicit smoking cessation behaviours among middle SES smokers who may be less responsive to tobacco taxation policies. In settings such as this with high HIV burden, HIV care infrastructure could be leveraged to provide tobacco cessation counseling and other interventions.

## Supporting information

S1 Table Covariate definitions. (DOCX)

S2 Table Pairwise correlation coefficients between covariates. (DOCX)

S3 Table Baseline characteristics of those who participated and did not participate in the follow-up survey. (DOCX)

S4 Table Sensitivity analysis: Logistic regression results showing variables associated with smoking cessation among baseline current smokers aged ≥15 years (N = 458) (when baseline current smokers who reported never smoking at follow-up are included in the denominator of the cessation variable). (DOCX)

S5 Table Sensitivity analysis: Logistic regression results showing variables associated with smoking relapse among baseline former smokers aged ≥15 years (N = 62) (when baseline former smokers who reported never smoking at follow-up are included in the denominator of the relapse variable). (DOCX)

S6 Table Sensitivity analysis: Logistic regression results showing variables associated with quitting smoking among baseline current smokers aged ≥15 years (N = 458) (when current smokers who reported never smoking are included in the numerator and denominator of the cessation variable). (DOCX)

S7 Table Vukuzazi Team: Staff who significantly contributed to the implementation and conduct of Vukuzazi. (DOCX)

S1 Fig Average adjusted predicted probabilities of smoking initiation among males and females aged 15–29 years. (DOCX)

S2 Fig Average adjusted predicted probabilities of smoking cessation by sex, socioeconomic status (SES) and smoking intensity. SES, socioeconomic status.
(DOCX)

S1 Checklist Questionnaire on inclusivity in global research. (DOCX)
