# Supplementary material for: Initiation, cessation and relapse of tobacco smoking over a 3-year period among participants aged ≥15 years in a large longitudinal cohort in rural South Africa
Source: PLOS Glob Public Health. 2025 Feb 25;5(2):e0004126. doi: 10.1371/journal.pgph.0004126 (PMC11856274; doi:10.1371/journal.pgph.0004126)
Supplement: S1 Table — (DOCX) [file pgph.0004126.s001.docx]

**S1 Table. Covariate definitions.**

| **Variable** | **Definition** |
| --- | --- |
| Sex | Biological sex: male or female. |
| Age at enrolment | Age at enrolment (years). |
| Socioeconomic status (SES) | Measured by an asset-based index derived from principal components analysis using a standard list of questions at baseline about household items, water source, toilet type and electricity source.^a^ The index was categorized into quintiles corresponding to low, medium and high SES. |
| Employment status | Derived from multiple questions on employment status at baseline. Categorized as unemployed, employed, not in labor force and unknown. |
| HIV care cascade status | Categorized as:   - HIV negative - HIV positive and uncontrolled (either undiagnosed, diagnosed and not in care, or in care and viral load [VL]≥400 copies/ml) - HIV positive and controlled (diagnosed, in care and VL<400 copies/ml).   The enzyme-linked immunosorbent assay (ELISA) was used to assess HIV status from participants’ blood specimens. |
| Years since started smoking (smoking duration) | Difference between the participant’s age at baseline and the reported age at which they started smoking. |
| Smoking intensity | Derived from the total numbers of combustible tobacco products used on a typical day reported at baseline. Categorized as light (<10 products per day), moderate (10-19) and heavy (>=20) smoking, where the latter two groups were combined due to the small numbers of heavy smokers with data on quitting outcomes. |
| Attempted to quit smoking (past 12 months) | Derived from baseline question: “During the past 12 months, have you tried to stop smoking?” – Yes or No. |
| Advised to quit smoking by a health care provider | Derived from baseline question: “During any visit to a doctor or other health worker in the past 12 months, were you advised to quit smoking tobacco?” – Yes, No, No visit to health provider (where the latter 2 categories were coded as “No”). |
| Consumed alcohol in past 12 months | Baseline consumption of any alcohol during the 12 months preceding the survey (such as beer, wine, spirits or other local alcoholic beverages). |
| Consumed alcohol in past 30 days | Baseline consumption of any alcohol during the 30 days preceding the survey (such as beer, wine, spirits or other local alcoholic beverages). |
| Hypertension | Having been on treatment for high blood pressure in the past 2 weeks or having a mean systolic blood pressure>=140 mmHg or mean diastolic blood pressure >=90 mmHg. |
| Diabetes | Having been on treatment for diabetes in the past 2 weeks or having an HbA1c>=6.5%. |
| Difficulties in daily activities | Self-reported difficulties in any of mobility, self-care, usual activities or pain problems and was used as a proxy for disability. |

1. Filmer, D. and L.H. Pritchett, Estimating Wealth Effects without Expenditure Data-or Tears: An Application to Educational Enrollments in States of India. Demography, 2001. 38(1): p. 115-132. 10.2307/3088292.
