# Supplementary material for: Initiation, cessation and relapse of tobacco smoking over a 3-year period among participants aged ≥15 years in a large longitudinal cohort in rural South Africa
Source: PLOS Glob Public Health. 2025 Feb 25;5(2):e0004126. doi: 10.1371/journal.pgph.0004126 (PMC11856274; doi:10.1371/journal.pgph.0004126)
Supplement: S2 Table — (DOCX) [file pgph.0004126.s002.docx]

**S2 Table. Pairwise correlation coefficients between covariates.**

|  | Incident TB | Sex | Attempted to quit smoking | Smoking intensity | Hypertension diagnosis | Diabetes diagnosis | Age | HIV care cascade status | Daily difficul-ties | Consumed alcohol (past 30 days) | Employment status | SES | Years since started smoking | Advised to quit smoking |
| --- | --- | --- | --- | --- | --- | --- | --- | --- | --- | --- | --- | --- | --- | --- |
| Incident TB | 1.000 |  |  |  |  |  |  |  |  |  |  |  |  |  |
| Sex | -0.043 | 1.000 |  |  |  |  |  |  |  |  |  |  |  |  |
| Attempted to quit smoking | -0.071 | 0.039 | 1.000 |  |  |  |  |  |  |  |  |  |  |  |
| Smoking intensity | 0.073 | 0.018 | 0.000 | 1.000 |  |  |  |  |  |  |  |  |  |  |
| Hypertension diagnosis | 0.055 | 0.027 | -0.031 | 0.051 | 1.000 |  |  |  |  |  |  |  |  |  |
| Diabetes diagnosis | 0.023 | 0.028 | -0.061 | -0.083 | 0.091 | 1.000 |  |  |  |  |  |  |  |  |
| Age at enrolment | -0.081 | -0.025 | 0.001 | 0.047 | -0.268 | -0.134 | 1.000 |  |  |  |  |  |  |  |
| HIV care cascade status | -0.103 | 0.029 | -0.064 | -0.009 | -0.033 | 0.103 | -0.006 | 1.000 |  |  |  |  |  |  |
| Daily difficulties | -0.118 | -0.068 | 0.017 | -0.022 | -0.051 | -0.060 | 0.107 | 0.095 | 1.000 |  |  |  |  |  |
| Consumed alcohol (past 30 days) | -0.016 | 0.031 | -0.098 | 0.144 | 0.016 | 0.020 | 0.017 | -0.055 | 0.032 | 1.000 |  |  |  |  |
| Employment status | -0.038 | 0.050 | -0.003 | -0.037 | 0.017 | 0.042 | 0.088 | -0.098 | -0.012 | 0.047 | 1.000 |  |  |  |
| SES | 0.110 | 0.081 | 0.085 | -0.007 | 0.037 | -0.020 | -0.073 | -0.078 | -0.023 | -0.066 | 0.028 | 1.000 |  |  |
| Years since started smoking | -0.073 | -0.099 | 0.058 | 0.065 | -0.064 | -0.039 | 0.161 | 0.103 | 0.075 | 0.029 | -0.084 | 0.026 | 1.000 |  |
| Advised to quit smoking | 0.036 | -0.047 | -0.224 | 0.014 | -0.005 | 0.037 | 0.034 | 0.061 | 0.073 | -0.064 | -0.042 | 0.048 | 0.003 | 1.000 |

SES: socioeconomic status, TB: tuberculosis.
