# Supplementary material for: Initiation, cessation and relapse of tobacco smoking over a 3-year period among participants aged ≥15 years in a large longitudinal cohort in rural South Africa
Source: PLOS Glob Public Health. 2025 Feb 25;5(2):e0004126. doi: 10.1371/journal.pgph.0004126 (PMC11856274; doi:10.1371/journal.pgph.0004126)
Supplement: S3 Table — (DOCX) [file pgph.0004126.s003.docx]

**S3 Table. Baseline characteristics of those who participated and did not participate in the follow-up survey.**

|  | Did not participate in follow-up survey | | Participated in follow-up survey | | p-value |
| --- | --- | --- | --- | --- | --- |
|  | % | count | % | count |  |
| Total | 100.0 | 694 | 100.0 | 754 |  |
| Sex |  |  |  |  | 0.022 |
| Male | 80.0 | 555 | 74.9 | 565 |  |
| Female | 20.0 | 139 | 25.1 | 189 |  |
| Age (median, IQR) | 31.0 | 22-44 | 30.5 | 23-47 | 0.825 |
| HIV status |  |  |  |  | 0.267 |
| Positive | 46.7 | 324 | 49.6 | 374 |  |
| Negative | 53.3 | 370 | 50.4 | 380 |  |
| Employment status |  |  |  |  | 0.203 |
| Employed | 21.9 | 152 | 22.3 | 168 |  |
| Unemployed | 50.3 | 349 | 51.5 | 388 |  |
| Not in labour force | 10.2 | 71 | 12.3 | 93 |  |
| Unknown | 17.6 | 122 | 13.9 | 105 |  |
| Socioeconomic status |  |  |  |  | 0.069 |
| Low | 46.4 | 312 | 40.3 | 295 |  |
| Middle | 21.7 | 146 | 23.5 | 172 |  |
| High | 31.9 | 215 | 36.2 | 265 |  |

IQR: interquartile range.
