# Supplementary material for: Initiation, cessation and relapse of tobacco smoking over a 3-year period among participants aged ≥15 years in a large longitudinal cohort in rural South Africa
Source: PLOS Glob Public Health. 2025 Feb 25;5(2):e0004126. doi: 10.1371/journal.pgph.0004126 (PMC11856274; doi:10.1371/journal.pgph.0004126)
Supplement: S4 Table — (DOCX) [file pgph.0004126.s004.docx]

**S4 Table. Sensitivity analysis: Logistic regression results showing variables associated with smoking cessation among baseline current smokers aged ≥15 years (N=458) (when baseline current smokers who reported never smoking at follow-up are included in the denominator of the cessation variable).**

|  | Univariate logistic regression | | | Multiple logistic regression | | |
| --- | --- | --- | --- | --- | --- | --- |
|  | OR | 95% CI(OR) | p-value | AOR | 95% CI(OR) | p-value |
| Sex |  |  |  |  |  |  |
| Male | 1.46 | [0.50-4.23] | 0.491 |  |  |  |
| Female | ref | - | - |  |  |  |
| Age at enrolment |  |  |  |  |  |  |
| 15-49 years | ref | - | - |  |  |  |
| ≥50 years | 1.16 | [0.62-2.17] | 0.643 |  |  |  |
| HIV care cascade status |  |  |  |  |  |  |
| Negative | ref | - | - |  |  |  |
| Positive and uncontrolled | 0.66 | [0.24-1.79] | 0.414 |  |  |  |
| Positive and controlled | 0.89 | [0.48-1.65] | 0.718 |  |  |  |
| Incident tuberculosis |  |  |  |  |  |  |
| No | ref | - | - |  |  |  |
| Yes | 2.11 | [0.87-5.13] | 0.098 | 1.82 | [0.73-4.55] | 0.201 |
| Daily difficulties |  |  |  |  |  |  |
| None | ref | - | - |  |  |  |
| Some | 0.75 | [0.28-1.98] | 0.561 |  |  |  |
| Consumed alcohol in past 30 days |  |  |  |  |  |  |
| No | ref | - | - |  |  |  |
| Yes | 0.99 | [0.52-1.89] | 0.969 |  |  |  |
| Employment |  |  |  |  |  |  |
| Employed | ref | - | - | ref | - | - |
| Unemployed | 0.98 | [0.50-1.94] | 0.956 | 0.87 | [0.43-1.75] | 0.691 |
| Not in labour force | 1.30 | [0.47-3.61] | 0.618 | 1.01 | [0.35-2.88] | 0.989 |
| Unknown | 3.16 | [1.06-9.40] | 0.038 | 3.28 | [1.07-10.07] | 0.038 |
| Socioeconomic status |  |  |  |  |  |  |
| Low | ref | - | - | ref | - | - |
| Middle | 0.48 | [0.21-1.12] | 0.088 | 0.48 | [0.21-1.13] | 0.094 |
| High | 0.78 | [0.41-1.48] | 0.449 | 0.77 | [0.40-1.51] | 0.453 |
| Hypertension |  |  |  |  |  |  |
| No | ref | - | - |  |  |  |
| Yes | 1.30 | [0.66-2.54] | 0.449 |  |  |  |
| Diabetes |  |  |  |  |  |  |
| No | ref | - | - |  |  |  |
| Yes | 0.63 | [0.08-4.93] | 0.658 |  |  |  |
| Smoking intensity at baseline |  |  |  |  |  |  |
| Light | ref | - | - | ref | - | - |
| Moderate to heavy | 0.42 | [0.15-1.22] | 0.112 | 0.34 | [0.10-1.16] | 0.085 |
| Unknown | 1.64 | [0.82-3.29] | 0.160 | 1.81 | [0.88-3.73] | 0.107 |
| Years since started smoking |  |  |  |  |  |  |
| 1-5 years | ref | - | - |  |  |  |
| >5 years | 0.76 | [0.34-1.67] | 0.491 |  |  |  |
| Unknown | 0.68 | [0.25-1.82] | 0.443 |  |  |  |
| Attempted to quit smoking in past 12 months |  |  |  |  |  |  |
| No | ref | - | - |  |  |  |
| Yes | 0.93 | [0.40-2.16] | 0.864 |  |  |  |
| Advised to quit smoking by a health care provider |  |  |  |  |  |  |
| No | ref | - | - |  |  |  |
| Yes | 0.62 | [0.14-2.71] | 0.528 |  |  |  |

OR: odds ratio, AOR: adjusted odds ratio, CI: confidence interval.
