# Supplementary material for: Initiation, cessation and relapse of tobacco smoking over a 3-year period among participants aged ≥15 years in a large longitudinal cohort in rural South Africa
Source: PLOS Glob Public Health. 2025 Feb 25;5(2):e0004126. doi: 10.1371/journal.pgph.0004126 (PMC11856274; doi:10.1371/journal.pgph.0004126)
Supplement: S5 Table — (DOCX) [file pgph.0004126.s005.docx]

**S5 Table. Sensitivity analysis: Logistic regression results showing variables associated with smoking relapse among baseline former smokers aged ≥15 years (N=62) (when baseline former smokers who reported never smoking at follow-up are included in the denominator of the relapse variable).**

|  | Univariate logistic regression | | | Multiple logistic regression | | |
| --- | --- | --- | --- | --- | --- | --- |
|  | OR | 95% CI(OR) | p-value | AOR | 95% CI(OR) | p-value |
| Age at enrolment |  |  |  |  |  |  |
| 15-49 years | ref | - | - | ref | - | - |
| ≥50 years | 0.45 | [0.07-2.91] | 0.403 |  |  |  |
| HIV status |  |  |  |  |  |  |
| Positive | ref | - | - | ref | - | - |
| Negative | 4.44 | [0.47-42.26] | 0.194 | 5.37 | [0.52-55.5] | 0.159 |
| Consumed alcohol in past 12 months |  |  |  |  |  |  |
| No | ref | - | - |  |  |  |
| Yes | 0.70 | [0.07-6.77] | 0.758 |  |  |  |
| Socioeconomic status |  |  |  |  |  |  |
| Low | ref | - | - | ref | - | - |
| Middle | 6.25 | [0.59-66.56] | 0.129 | 8.02 | [0.7-92.00] | 0.094 |
| High | 1.32 | [0.08-22.41] | 0.850 | 1.58 | [0.09-27.92] | 0.754 |

OR: odds ratio, AOR: adjusted odds ratio, CI: confidence interval.
