# Supplementary material for: Initiation, cessation and relapse of tobacco smoking over a 3-year period among participants aged ≥15 years in a large longitudinal cohort in rural South Africa
Source: PLOS Glob Public Health. 2025 Feb 25;5(2):e0004126. doi: 10.1371/journal.pgph.0004126 (PMC11856274; doi:10.1371/journal.pgph.0004126)
Supplement: S6 Table — (DOCX) [file pgph.0004126.s006.docx]

**S6 Table. Sensitivity analysis: Logistic regression results showing variables associated with quitting smoking among baseline current smokers aged >=15 years (N=458) (when current smokers who reported never smoking are included in the numerator and denominator of the cessation variable).**

|  | Univariate logistic regression | | | Multiple logistic regression | | |
| --- | --- | --- | --- | --- | --- | --- |
|  | OR | 95% CI(OR) | p-value | AOR | 95% CI(OR) | p-value |
| Sex |  |  |  |  |  |  |
| Male | 0.05 | [0.02-0.10] | <0.001 | 0.05 | [0.02-0.11] | <0.001 |
| Female | ref | - | - | ref | - |  |
| Age at enrolment |  |  |  |  |  |  |
| 15-49 years | ref | - | - |  |  |  |
| ≥50 years | 1.17 | [0.72 - 1.90] | 0.529 |  |  |  |
| HIV care cascade status |  |  |  |  |  |  |
| Negative | ref | - | - |  |  |  |
| Positive and uncontrolled | 0.62 | [0.29 - 1.35] | 0.227 |  |  |  |
| Positive and controlled | 0.99 | [0.61 - 1.59] | 0.958 |  |  |  |
| Incident tuberculosis |  |  |  |  |  |  |
| No | ref | - | - |  |  |  |
| Yes | 1.10 | [0.48 - 2.51] | 0.820 |  |  |  |
| Daily difficulties |  |  |  |  |  |  |
| None | ref | - | - |  |  |  |
| Some | 1.02 | [0.51 - 2.01] | 0.963 |  |  |  |
| Consumed alcohol in past 30 days |  |  |  |  |  |  |
| No | ref | - | - | ref | - | - |
| Yes | 0.63 | [0.37 - 1.08] | 0.091 | 0.93 | [0.49 - 1.77] | 0.836 |
| Employment |  |  |  |  |  |  |
| Employed | ref | - | - | ref | - | - |
| Unemployed | 1.35 | [0.78 - 2.33] | 0.284 | 0.75 | [0.39 - 1.41] | 0.369 |
| Not in labour force | 1.82 | [0.81 - 4.06] | 0.145 | 1.09 | [0.43 - 2.74] | 0.854 |
| Unknown | 5.00 | [1.93 - 12.97] | 0.001 | 4.47 | [1.43 - 13.91] | 0.010 |
| Socioeconomic status |  |  |  |  |  |  |
| Low | ref | - | - | ref | - | - |
| Middle | 0.37 | [0.20 - 0.72] | 0.003 | 0.33 | [0.16 - 0.72] | 0.005 |
| High | 0.62 | [0.38 - 1.03] | 0.067 | 0.59 | [0.32 - 1.08] | 0.088 |
| Hypertension |  |  |  |  |  |  |
| No | ref | - | - | ref | - | - |
| Yes | 1.39 | [0.82 - 2.35] | 0.216 | 1.42 | [0.75 - 2.72] | 0.285 |
| Diabetes |  |  |  |  |  |  |
| No | ref | - | - | ref | - | - |
| Yes | 2.30 | [0.73 - 7.18] | 0.153 | 1.83 | [0.31 - 10.70] | 0.502 |
| Smoking intensity at baseline |  |  |  |  |  |  |
| Light | ref | - | - | ref | - | - |
| Moderate to heavy | 0.22 | [0.09 - 0.56] | 0.002 | 0.21 | [0.06 - 0.66] | 0.008 |
| Unknown | 1.18 | [0.66 - 2.10] | 0.579 | 1.42 | [0.72 - 2.82] | 0.317 |
| Years since started smoking |  |  |  |  |  |  |
| 1-5 years | ref | - | - | ref | - | - |
| >5 years | 0.40 | [0.22 - 0.73] | 0.002 | 0.76 | [0.36 - 1.61] | 0.478 |
| Unknown | 0.44 | [0.21 - 0.92] | 0.029 | 0.66 | [0.26 - 1.65] | 0.375 |
| Attempted to quit smoking in past 12 months |  |  |  |  |  |  |
| No | ref | - | - |  |  |  |
| Yes | 0.80 | [0.41 - 1.57] | 0.520 |  |  |  |
| Advised to quit smoking by a health care provider |  |  |  |  |  |  |
| No | ref | - | - |  |  |  |
| Yes | 0.64 | [0.21 - 1.89] | 0.416 |  |  |  |

OR: odds ratio, AOR: adjusted odds ratio, CI: confidence interval.
