# Supplementary material for: Initiation, cessation and relapse of tobacco smoking over a 3-year period among participants aged ≥15 years in a large longitudinal cohort in rural South Africa
Source: PLOS Glob Public Health. 2025 Feb 25;5(2):e0004126. doi: 10.1371/journal.pgph.0004126 (PMC11856274; doi:10.1371/journal.pgph.0004126)
Supplement: S1 Fig — (DOCX) [file pgph.0004126.s008.docx]

**S1 Fig. Average adjusted predicted probabilities of smoking initiation among males and females aged 15-29 years.**
